# Characterization of the microbiome and volatile compounds in anal gland secretions from domestic cats (Felis catus) using metagenomics and metabolomics

**DOI:** 10.21203/rs.3.rs-2883555/v1

**Published:** 2023-05-09

**Authors:** Connie A. Rojas, Stanley L. Marks, Eva Borras, Hira Lesea, Mitchell M. McCartney, David Coil, Cristina E. Davis, Jonathan A. Eisen

**Affiliations:** University of California – Davis

## Abstract

Animals rely on volatile chemical compounds for their communication and behavior. Many of these compounds are sequestered in endocrine and exocrine glands and are synthesized by anaerobic microbes. While the volatile organic compound (VOC) or microbiome composition of glandular secretions has been investigated in several mammalian species, few have linked specific bacterial taxa to the production of volatiles or to specific microbial gene pathways. Here, we use metagenomic sequencing, mass-spectrometry based metabolomics, and culturing to profile the microbial and volatile chemical constituents of anal gland secretions in twenty-three domestic cats (*Felis catus*), in attempts to identify organisms potentially involved in host odor production. We found that the anal gland microbiome was dominated by bacteria in the genera *Corynebacterium, Bacteroides, Proteus, Lactobacillus*, and *Streptococcus*, and showed striking variation among individual cats. Microbiome profiles also varied with host age and obesity. Metabolites such as fatty-acids, ketones, aldehydes and alcohols were detected in glandular secretions. Overall, microbiome and metabolome profiles were modestly correlated (r=0.17), indicating that a relationship exists between the bacteria in the gland and the metabolites produced in the gland. Functional analyses revealed the presence of genes predicted to code for enzymes involved in VOC metabolism such as dehydrogenases, reductases, and decarboxylases. From metagenomic data, we generated 85 high-quality metagenome assembled genomes (MAGs). Of these, four were inferred to have high relative abundance in metagenome profiles and had close relatives that were recovered as cultured isolates. These four MAGs were classified as *Corynebacterium frankenforstense, Proteus mirabilis, Lactobacillus johnsonii*, and *Bacteroides fragilis*. They represent strong candidates for further investigation of the mechanisms of volatile synthesis and scent production in the mammalian anal gland.

## Introduction

Many amniotes (reptiles, birds, and mammals) communicate using chemical cues; cues that govern their social behavior, development, reproduction, and intra- and inter-specific interactions [1-4]. These chemical compounds include volatile organic compounds (VOCs) found in feces, urine, or saliva [5], and in glandular secretions [1, 6-9]. These volatile compounds can assist with individual recognition [10, 11], kin recognition [12-14], territory defense [15, 16], reproductive advertisement [17, 18], mate attraction [19, 20], and can even deter predators [21, 22] or reduce the spread of parasites [23]. Animal glandular secretions in particular can signal and encode information about the individual identity [24], age [25], sex [26, 27], reproductive status [19, 28], health condition [29], social status [30, 31] and social group [32, 33] of the sender. While mammalian hosts have proteins (e.g. mammalian aldehyde dehydrogenases, fatty acid synthases, and odorant binding proteins) that can bind, synthesize, or transport VOCs that are a constituent of scent gland secretions [34-36], the majority of volatiles are actually produced by fermentative bacteria that reside within the gland itself. Several studies have examined the volatile or microbiome profiles of glandular secretions from a variety of species [27, 33, 37-39], but few have linked specific bacterial taxa to the *in vitro* production of volatiles or to specific microbial gene pathways [37, 38] that may underlie the production of those volatiles.

Many scent-producing mammals have evolved skin-derived glands to produce, store, and disseminate odorous volatile compounds [40], which include aldehydes, amides, alkanes, aromatics, alcohols, hydrocarbons, fatty acids, esters, ketones, phenols, squalenes, and steroids [41]. Prior surveys on the bacterial taxonomic composition of glandular microbiomes in primates [42], carnivores [27], and bats [43] provide hints as to which bacteria might be involved in the production of volatile compounds found within these glands [41]. In the giant panda [38], the most abundant bacterial genera of the anogenital gland – a cutaneous gland in the anogenital region – are *Corynebacterium, Pseudomonas, Porphyromonas, Psychrobacter*, and *Anaerococcus*. In the tail subcaudal gland secretions of owl monkeys, *Bacillus, Staphylococcus, Enterococcus, Lactococcus, Paenibacillus, Proteus*, and *Pseudomonas have* all been recovered [43]. The anal glands of domestic dogs – situated between the internal and external anal sphincter muscles – mainly contain species from the bacterial genera *Enterococcus, Bacteroides*, and *Proteus* [44]. In lesser long-nosed bats, adult males develop a dorsal patch in the interscapular region to attract females during the breeding season [45, 46], and several bacterial groups in this patch are hypothesized to be involved in its odor production (*Gallicola, Anaerococcus, Peptoniphilus, Proteus*, and *Clostridium*).

A few experimental studies have found more direct links between specific bacterial species and the production of odor compounds in scent-producing glands. In dark-eyed juncos, hoopoes, and mongooses, treating glandular secretions with antibiotics changes the composition of the microbial community and inhibits the production of volatile compounds [47-49]. Experimental work shows that *Bacillus* sp. can use glucose and threonine as substrates to produce volatile compounds that function as pheromones in oriental flies [50]. Most recently, a metagenomic survey of giant panda anogenital gland secretions found that several metabolic pathways likely involved in the production of VOCs were overrepresented in glandular secretions compared to feces [38]. Among those pathways were fatty acid biosynthesis, the synthesis and degradation of ketone bodies, steroid biosynthesis, the biosynthesis of unsaturated fatty acids, and ether lipid metabolism. The genes and pathways primarily came from bacteria in the *Nocardiaceae, Micrococcaceae, Corynebacteriaceae*, and *Desulfobacteraceae* families [38]. However, this type of information is largely unknown for other carnivores, especially felids. Here, we address some of these gaps in knowledge and examine the bacterial and volatile chemical composition of anal gland secretions in domestic cats (*Felis cactus*), and identify possible bacterial species and pathways via which these odorant molecules may be produced.

In domestic cats, anal glands produce both apocrine and sebaceous secretions that emit strong pungent odors [51, 52]. A prior chemical analysis found that the major odor compounds in the anal gland were short-chain free fatty acids including acetic acid, propanoic acid, 2-methylpropanoic acid, butanoic acid, 3-methylbutanoic acid, and pentanoic acid. Metabolite profiles varied among individuals but not among sexes [53]. Although the bacterial taxonomic composition of the anal glands of true domestic cats has not yet been examined, in the Bengal cat (*Felis catus × Prionailurus bengalensis*) three microbes isolated from the anal gland produce many of the same chemical volatiles found in the secretions [37]. These three microbes – *Bacteroides fragilis, Tessarococcus* spp., and *Finegoldia magna* – were also relatively abundant in the accompanying 16S rRNA gene sequence data. The combined evidence from these two prior studies suggests that several bacterial species in the felid scent gland likely have the metabolic machinery to synthesize odorant molecules that are employed by their host during chemical communication.

In this study, we build on these findings and use culture-dependent techniques in combination with shotgun metagenomics and metabolomics (thermal desorption-gas chromatography-mass spectrometry, TD-GC-MS) to examine anal gland secretions in twenty-three companion cats (N = 23; Table 1) that were evaluated at a tertiary referral hospital. Some of these cats had been previously diagnosed with periodontal disease, chronic enteropathy, or renal or intranasal disease but were asymptomatic and clinically healthy at the time of sampling. More importantly, their anal glands were healthy and free of abscesses, clogs, or infections. In this study, we profile the bacterial and volatile components of anal gland secretions, and determine whether differences are found among cats of distinct ages, body conditions (obese vs. not), living environments, diets, or a medical diagnosis of periodontitis, which is characterized by plaque buildup and inflammation of the gums. Furthermore, we report the extent to which microbiome and VOC profiles are correlated and examine microbiome functions to identify metabolic pathways potentially involved in VOC synthesis. Lastly, we reconstructed metagenome-assembled genomes (MAGs) from shotgun sequence data which can be further studied and annotated to advance our understanding of the microbes involved in host chemical communication.

## Methods

Sampling the anal gland and perianal region of domestic cats. Companion cats (N = 23) that presented to the Veterinary Medical Teaching Hospital at UC Davis between December 2021 and March 2022 for elective procedures were evaluated for their suitability for enrollment in the study. The elective procedures included dental cleaning, abdominal ultrasounds, radiographs, or oral examinations that required sedation or general anesthesia. Sedation or general anesthesia of companion cats at the UCD Veterinary Medical Teaching Hospital is carried by anesthesiologists and clinicians using established protocols (e.g. Dexmedetomidine at 4mcg/kg IV and Methadone at 0.2mg/kg IV for sedation, or Maropitant at 1 mg/kg IV and Butorphanol at 0.2–0.4 mg/kg IM for anesthesia). After obtaining informed written consent from cat owners, a board-certified internist and gastroenterologist (S.L.M) manually expressed the anal glands of cats while they were sedated by inserting a lubricated gloved index finger into the cat’s anus and digitally squeezing the anal gland between the index finger and thumb into a sterile 2” x 2” gauze sponge. Collected anal gland material was immediately transferred onto three sterile Puritan cotton swabs (one for microbiome analysis, one for microbial culturing, and one for metabolomics). In addition, a swab from the cat’s perianal region was collected for comparison to the microbiome found in the anal gland. Finally, an unused Puritan cotton swab (blank negative control) and a swab of the internist’s examination glove prior to the procedure were collected as well to assess background contamination.

The swabs were placed in 2 mL screw cap tubes (for microbiome or culturing analysis) or 20 mL borosilicate glass vials (for metabolomic analysis) and stored at −80°C until laboratory and chemical analysis. After sample collection, the cat’s perianal region was cleaned with warm water and sprayed with a feline deodorizer. The cat was closely monitored and observed during recovery. Data on the animal’s signalment (age, breed, sex), health status (including any medical diagnoses), lifestyle (living environment), body weight, body condition, and diet was also collected (**Table S1**). At the time of sample collection, none of the cats had diarrhea and although some had been previously diagnosed with periodontal disease, chronic enteropathy, renal disease, or intranasal disease, they were asymptomatic for these conditions and deemed overall healthy. With the exception of a single cat, individuals had not been treated with systemic antibiotics within 6-months of enrollment in the study. We kept this cat in our dataset as its microbiome was not compositionally anomalous in any obvious way compared to those from other surveyed cats.

The study was approved by the University of California, Davis, Institutional Animal Care and Use Committee (IACUC protocol # 22528). All methods were performed in accordance with the relevant guidelines and regulations, including the ARRIVE guidelines.

Bacterial culturing and isolation from the anal gland. Bacterial cultures of the anal gland were obtained from 11 of the 23 cats (**Table S1**); we did not culture microbes from the remaining 12 cats due to timing. Bacterial swabs from the anal gland were vortexed with 1 mL of PBS and two serial 1:10 dilutions were performed. For each mixture, 150 μL was pipetted into lysogeny broth (LB), brain heart infusion (BHI), and blood agar (BA) plates. The plates were placed in BD GasPak EZ Anaerobic System boxes (BD Biosciences, NJ, USA) with packets of CO_2_ generators to maintain an anaerobic environment. After growing plates at 37°C for 3–5 days, colonies with distinct morphologies, colors, and textures were picked from any or all of the three types of agar plates and plated until pure colonies were obtained. Single colonies were then added to 5 mL of sterile LB, BHI, or BA liquid broth, degassed with nitrogen to remove any oxygen, and grown for three days at 37°C in preparation for DNA extractions.

DNA extraction and Sanger sequencing of bacterial cultures. DNA was extracted from liquid bacterial broth cultures using the Wizard SV Genomic DNA Purification Kits (Promega, USA), according to their protocol for Gram-negative bacteria. Briefly, 1.5 mL of vortexed broth was centrifuged and subsequently exposed to nuclei lysis, RNAse digestion, incubation at 37°C, and protein precipitation on ice. The DNA was washed with isopropanol and ethanol, and the pellet was air-dried and then resuspended in nuclease-free water.

For each bacterial isolate, the 16S rRNA gene was amplified using the 27F (5'-AGAGTTTGATCMTGGCTCAG-3') and 1391R (5'-GACGGGCGGTGTGTRCA-3') bacterial-specific primers. The PCR conditions were as follows: an initial denaturation step at 95°C for 3 mins, followed by 30 cycles of 95°C for 45 seconds, 50°C for 60 seconds and 72°C for 90 seconds. A final extension occurred at 72°C for 10 minutes, and a final hold at 15°C. PCR products were purified with the NucleoSpin Gel and PCR Clean-Up kit (Takara Bio, CA, USA) and quantified with Qubit HS dsDNA assay (Thermo Scientific, MA, USA). PCR products were diluted with nuclease-free water until achieving a concentration of ~ 32 ng/μL in preparation for Sanger sequencing. Samples with initial concentrations between 10–35 ng/μL were not diluted, and those with concentrations < 10 ng/μL were not sequenced.

A total of 111 bacterial isolates were submitted for Sanger sequencing of the 16S rRNA gene (27F primer) at the UC Davis College of Biological Sciences DNA Sequencing Facility (Davis, CA, USA). Sanger chromatograms were uploaded to myRDP [54] for quality-trimming and base-calling. The trimmed sequences were searched against the bacterial NCBI RefSeq genomes database [55] using blastn for taxonomic identification setting default parameters. The top hit with the highest e-value and percent identity was selected as that organism’s taxon label (**Table S18**). Six of the 111 isolates did not meet sequence quality thresholds and could not be classified taxonomically.

DNA extraction and metagenomic sequencing of bacterial swabs. Genomic DNA was extracted directly from swabs of the anal gland (N = 23), perianal region (N = 6), and controls (a sterile swab and swab of glove; N = 2) using the QIAGEN DNeasy Powersoil Pro Kits (Qiagen, MD, USA) (**Table S1**). Swabs were incubated with the QIAGEN CD1 lysis buffer (800 mM guanidine hydrochloride; 30 mM Tris•Cl, pH 8.0; 30 mM EDTA, pH 8.0; 5% Tween 20; 0.5% Triton X-100) at 65°C for 10 minutes and then underwent bead-beating for 1.5 mins before resuming the manufacturer’s protocol for this kit. Genomic DNA from 31 samples was treated with RNAse A and sequenced on an Illumina NextSeq 500 (300 cycles; Illumina, CA, USA) at the UC Davis Genomics Core to generate PE x 150 bp reads.

The general protocol used by the UC Davis Genome Core for this work was as follows: briefly, barcode-indexed sequencing libraries were generated from genomic DNA samples sheared on an E220 Focused Ultrasonicator (Covaris, Woburn, MA). For each sample 1-130 ng sheared DNA was converted to sequencing libraries using a Kapa High Throughput Library Preparation Kit (Kapa Biosystems-Roche, Basel, Switzerland). The libraries were amplified with 5–15 PCR cycles and analyzed with a Bioanalyzer 2100 instrument (Agilent, Santa Clara, CA), quantified by fluorometry on a Qubit instrument (LifeTechnologies, Carlsbad, CA), and combined in one pool at equimolar ratios. The pools were quantified by qPCR with a Kapa Library Quant kit (Kapa Biosystems-Roche) and subsequently sequenced.

Sequence processing of metagenomic data. Sequenced samples had an average of 5,527,421 (± 997,985) metagenomic paired-end reads which were quality-filtered and trimmed using Trimmomatic v.0.38 setting default parameters [56]. On average, samples retained 95% (± 1.07%) of their sequences after quality-filtering (**Table S2**). Reads were filtered of host DNA by aligning them to two *Felis catus* reference genomes (GenBank accessions GCA_000181335.5 & GCA_013340865.1) using Bowtie2 [57]. Fifteen samples retained > 92% of their quality-filtered sequences after removal of host DNA, another four samples retained 62–75% of their sequences, and six samples retained < 50% of their sequences (**Table S2**). For samples from the perianal region, three retained 50–70% of reads after host DNA filtering, while the other three only retained 12–15% of their reads. Kraken 2 [58] assigned taxonomic classification to the host-filtered quality-trimmed reads for each sample and Bracken (Bayesian Reestimation of Abundance with Kraken) [59] estimated the taxon abundances at the Family, Genus, and species level. On average, 55% of reads were classified by Kraken2 for each sample (range: 26–86%) (**Table S2**). When Kraken was not able to assign a species label to a sequence, it used the genus or family label followed by spp. (e.g. *Bacteroides* spp.).

The resulting forward and reverse metagenomic reads were interleaved using a python script from the Ray assembler v.2.3.1 [60] and concatenated into a single file in preparation for metagenome assembly. Sequences were assembled into contigs with (meta)SPAdes v.3.14.1 [61] and the quality of assembly was evaluated with QUAST v.5.0.0 [62] (**Table S3**). After removing contigs shorter than 300bp using BBMap v.38.87, contigs were uploaded into Anvi’o v.7.1 [63] for gene prediction and functional annotation. Gene prediction was accomplished with Prodigal v.2.6.3 [64], and functional annotation was done using the Clusters of orthologous groups (COGs) [65], and the Kyoto Encyclopedia of Genes and Genomes (KEGG) databases [66]. To determine the abundances of genes in each sample, host-filtered reads were mapped to predicted genes using Salmon (v1.8.0) [67]. Gene relative abundances were in units of Transcripts Per Million (TPM), which normalizes for both gene length and sample sequencing depth. On average, 70.35% of host-filtered reads mapped to putative ORFs (range: 46–79%) (**Table S2**). Lesser percentages were assigned KEGG or COG annotation (~ 46% of mapped reads).

Contigs with a minimum length of 600 bp were binned into metagenome-assembled genomes (MAGs) using MetaBat2 v.2.15 [68]. A total of 85 high-quality MAGs were recovered with completeness scores > 80% and contamination scores < 5% as assessed by CheckM (v1.1.3) [69] (**Table S4**). The Genome Taxonomy Database Toolkit (GTDB-Tk) (v1.5.0) [70] was used to assign taxonomic identity to MAGs using database release 202 [71]. The abundance of each MAG in a sample was estimated using CoverM (v0.6.1) (https://github.com/wwood/CoverM) by mapping interleaved host-filtered reads to each MAG (**Table S5**). On average, 60.37% of reads in each sample were able to be mapped to MAGs (range: 32–88%) (**Table S2**). A phylogeny of these MAGs was constructed with RAxML [72] using the multiple-sequence alignments generated by GTDB-Tk. We had no outgroup and instead rooted our tree to the only member of the phylum *Synergistota (Fretibacterium)*.

Extraction and analysis of anal gland metabolites. Volatile compounds were extracted from anal swab samples using two techniques. First, solid phase microextraction (SPME) fibers (50/30 μm DVB/CAR/PDMS coating) were exposed to swabs in vials to extract VOCs from the headspace. Then, VOCs were extracted from swabs using liquid phase extraction with methanol, followed by a derivatization process before being directly analyzed.

For headspace technique, a 1 μL aliquot of 10 mL/L decane-d22 was added to the 20 mL borosilicate glass vials containing the swabs as an internal standard. Two previously conditioned SPME fibers were exposed to anal swabs for 24 hours at room temperature, then capped and placed in a −20°C freezer until spectrometric analysis. For the liquid phase extraction, these same swabs were placed into 20 mL of methanol for 24 hours at room temperature to extract VOCs into solution. A 2 mL aliquot of each extract was transferred into a new vial and completely dried under nitrogen. Dried extracts were subsequently derivatized by adding 50 μL MTBSFTA (N-tert-Butyldimethylsilyl-N-methyltrifluoroacetamide with 1% tert-Butyldimethylchlorosilane) and 50 μL acetonitrile. Reconstituted samples were left to react for 1 hour at 60°C and stored at −20°C until spectrometric analysis.

VOCs from SPME fibers and derivatized extracts were analyzed with gas chromatography-mass spectrometry. For SPME fibers, the fiber was inserted into the inlet of an Agilent 6890N gas chromatograph (Agilent Technologies Inc.) set to 260°C. VOCs were desorbed from the fiber for 5 min in splitless mode while the GC oven was held at 40°C. For liquid extracts, 1 μL of each sample was injected into the GC inlet held at 260°C. For both samples, the oven was ramped to 120°C at 5°C/min, and then ramped to 280°C at 15°C/min, holding for 10 minutes. VOCs were separated on a DB-5ms column (30 m × 250 μm × 0.25 μm, Agilent Technologies Inc.) with a 1 mL/min constant flow of helium. Compounds were eluted through a 300°C transfer line into an Agilent 5795C mass spectrometer, which scanned 50 to 500 m/z with its source set to 230°C and quadrupole to 150°C.

Samples and blanks/controls were injected in a random order to produce reliable data. A standard Grob mixture was injected in triplicate to monitor instrument performance, and a standard mix of C_8_-C_30_ alkanes was analyzed to calculate the Kovats Retention Indices of each VOC. Raw data were first checked for qualitative reasons using Agilent’s Mass Hunter Qualitative Analysis B.06.00 software. GC-MS data were then deconvoluted and aligned using the recursive feature extraction on Profinder (Version B.08.00, Agilent Technologies Inc.) and Mass Profiler Professional (MPP, V13.0). An initial table was then obtained containing all peak intensities (rows) from each sample (column). Peaks from contaminants like siloxanes (base peaks 207, 221 and 281 m/z). Features that appeared in blanks with a signal more than five times the signal from samples (peak sample/blank ratio) were removed. These blanks were composed of: system blanks (instrumental blank without injection), Twister^®^ blanks (injection of clean twisters), blank vials and blank cotton swabs; the latter two were treated as if they were biological samples and underwent SPME extraction and liquid extraction as described above.

Compounds were tentatively identified by matching the mass spectra with structures available in the National Institute of Standards and Technology (NIST) 2020 Library and by matching calculated retention times with those reported in the literature.

Statistical analysis of microbiome and metabolome data. Unless otherwise stated, all sequence data was analyzed and visualized using the R statistical software program (v3.6.2) [73, 74]. Prior to any statistical analysis, we used R decontam to identify and remove contaminant bacterial taxa from the Kraken2 dataset based on their prevalence in control samples compared to samples from the anal gland and perianal region. A total of 58 bacterial species (**Table S6**) were deemed contaminants by decontam (had scores below the specified threshold of 0.5) and were removed. Read counts assigned to *“Homo”* were also removed.

The composition of bacterial communities from the felid anal gland (or the perianal region) was visualized using data generated by Kraken2/Bracken (**Table S7, Table S8**). For this, bar plots showing the relative abundances of bacterial families and genera were constructed using ggplot2 (v3.3.6) [75]. Next, we examined whether five host factors of interest: age (in yrs), obesity (obese vs. not obese), living environment (indoor vs. indoor & outdoor), diet (dry food only vs. other diets), and a medical condition (moderate to severe periodontal disease vs. no disease) could account for any of the variance in anal gland microbiome beta-diversity. For analyses, cats with body condition scores (BCS) 8–9 were considered “obese” and all other cats were classified as “not obese” [76]. This classification was selected because of its clinical relevance and the multitude of obesity-associated comorbidities.

Genus-level abundance data generated by Kraken2/Bracken was converted to presence/absence (for Jaccard dissimilarity), proportions (for Bray-Curtis dissimilarity), or applied a Center-Log-Ratio (clr) transformation (for Aitchinson dissimilarity). Permutational multivariate analyses of variance (PERMANOVAs) tested whether microbiome beta-diversity varied with the five host factors. Tests evaluated all factors simultaneously, in a way where the order of terms did not influence statistical output (e.g. they were marginal PERMANOVA tests). PERMANOVAs employed 999 permutations, set alpha to 0.05, and were conducted with the vegan package (v2.6-2) [77].

We investigated whether microbiome profiles from the anal gland were significantly correlated with metabolome profiles (from solid-phase and liquid-derivatization extractions). For this, the matrix of metabolite absolute abundances (**Table S9** for solid extraction, **Table S10** for liquid extraction) were normalized by converting to relative abundances (e.g. proportions), log_10_-transformed to minimize the influence of heteroscedasticity, and scaled with pareto scaling, which is advised for metabolite data [78]. Metabolite Jaccard and Euclidean distances were estimated using the phyloseq package. Mantel tests correlated microbiome matrices (Jaccard, Bray-Curtis and Aitchison) to metabolite Jaccard or Euclidean distances using 999 permutations. Because a statistically significant relationship existed between the two datasets, we plotted this relationship; specifically, we extracted the first two principal coordinates from metabolite and microbiome dissimilarity matrices using the cmdscale function from the stats package [73, 74]. Scatterplots showcased the relationship visually.

We also tested whether the relative abundances of bacterial species were significantly associated with the relative abundances of specific metabolites using Spearman correlations (R stats package). P-values were adjusted for multiple comparisons using FDR. Only bacterial species found in at least 90% of anal gland samples and at a mean relative abundance > 0.2% were considered. For metabolites, all 37 putatively identified metabolites were considered, along with 187 unidentified metabolites that had normalized and scaled mean relative abundances > 0.

Analyses on COG and KEGG ortholog and pathway abundances were also conducted (**Table S11-S14**). Gene abundances which were estimated in TPM were normalized using total sum scaling. PERMANOVAs examined whether microbiome functions were significantly predicted by host factors, using the methods described above. Mantel tests evaluated whether microbiome functional profiles were correlated with metabolite abundance data using Mantel tests as described for the microbiome-metabolite correlations.

All of the methods described previously in this section were also used to analyze metagenome-assembled genomes (MAG) abundances. PERMANOVAs tested whether MAG relative abundances (**Table S5**) or their presence / absence were significantly associated with host age, obesity, living environment, diet, and a medical diagnosis of periodontitis. Mantel tests evaluated whether MAG profiles were associated with metabolite profiles. Spearman correlations were used to correlate the abundances of all MAGs classified to species level (47 MAGs) to the abundances of 37 putatively identified metabolites and 193 unidentified metabolites.

Lastly, marginal PERMANOVA tests were also employed to test whether the taxonomic and functional compositions of microbiomes in the anal gland were distinct from those in the perianal region.

## Results

Characteristics of feline study participants. The bacterial and metabolite composition of the anal gland microbiomes of 23 cats were surveyed for this study; five of these cats also had corresponding metagenome sequences from the perianal region (**Table S1**). Cat participants were predominantly indoor (70%) domestic shorthairs (83%), ranging in age from 2–14 years old, with a median age of 6.9 years. (Table 1, **Table S1**). Fifty-two percent of participants were females and 48% were males. Seventeen percent of cats were underweight (BCS < 5), 43% were of healthy weight (BCS 5–6), and 40% were overweight or obese (BCS 7–9) (Table 1, **Table S1**). Over half of the surveyed cats (57%) were only fed dry kibble, 26% were fed canned food and dry kibble, and 17% had other diets. Thirty-nine percent of study participants had been previously diagnosed with moderate to severe periodontal disease and 13% were diagnosed with chronic enteropathy (IBD or intestinal lymphoma), though at the time of sampling, all cats were asymptomatic and appeared otherwise clinically healthy on physical examination. None of the cats had diarrhea when they were evaluated and all had healthy anal glands. One cat had been administered systemic antibiotics within the six months leading up to sample collection.

Which bacterial taxa inhabit the anal gland in cats? One key goal of this study was to survey the taxonomic, functional, and chemical composition of the anal gland microbiome in twenty-three domestic cats (Table 1), and in doing so, shed light on the bacteria or bacterial gene pathways potentially involved in the synthesis of volatile organic compounds (VOCs) likely being used by their host for chemical communication. The first step in this was to provide a snapshot of the microbiome composition of anal gland secretions.

**Figure 1:**
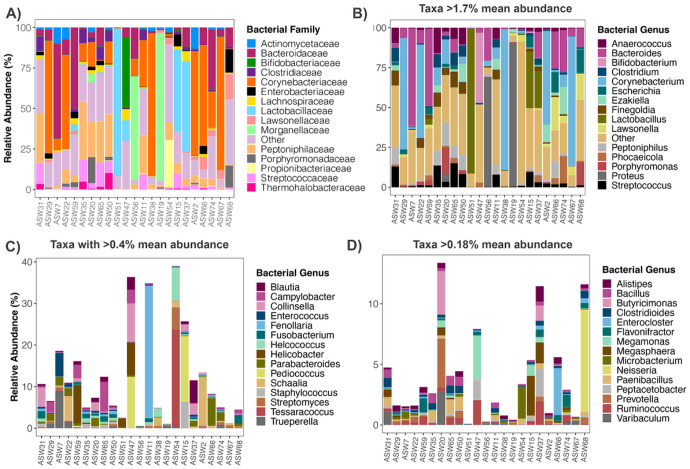
Bacterial taxonomic composition of the anal gland microbiome in domestic cats. Relative abundances of bacterial **A)** families and **B-D)**genera in anal gland metagenomes, as estimated from shotgun Illumina sequence data using Kraken2/Bracken. Families with a mean relative abundance >1% across samples are displayed while all others are collapsed into an “Other” category. For the remaining panels: Genera with a mean relative abundance >1.7% are shown in **B**, genera with mean relative abundances >0.4% but less than 1.7% are shown in **C**, and genera with mean relative abundances >0.18% but less than 0.4% are shown in **D**. We did this to showcase bacterial genera that were highly abundant in some samples, but not in all samples. Some of the genera collapsed under “Other” in panel B are shown in panels C-D.

The majority of metagenomic sequences recovered from the anal gland were classified as Bacteria (94% mean relative abundance) by the software Kraken2/Bracken, with the remainder of sequences classified as Eukaryotes (5% mean relative abundance), and Archaea and Viruses (< 1% combined mean relative abundance). The most represented bacterial families in feline anal glands included *Corynebacteriaceae* (23% mean relative abundance), *Bacteroidaceae* (11%), *Peptoniphilaceae* (10%), and *Lactobacillaceae* (9%) (Fig. 1A, **Table S7**). The single most frequently detected bacterial genus in sequencing reads was *Corynebacterium*, which on average constituted 18% of the microbiome in the anal gland. *Bacteroides* (11% mean relative abundance), *Proteus* (7%), *Lactobacillus* (7%), *Streptococcus* (4%), and *Peptoniphilus* (4%) had moderately high relative abundances (Fig. 1B, **Table S8**).

The microbiome compositions of cats were highly variable among individuals. The anal gland microbiomes of three cats for example was almost exclusively composed of *Corynebacterium* (> 70% relative abundance), while the anal gland microbiomes of other cats were dominated by *Proteus* (> 70% relative abundance) or *Bacteroides* (25–60% relative abundance) (Fig. 1B). The anal gland of one cat almost exclusively contained *Lactobacillus johnsonii* (88% relative abundance), and in other samples, this same bacterial taxon was rare (< 3% relative abundance). Interestingly, the microbiome of the cat that received antibiotics (sample ID ASW22) was compositionally similar to that of a cat of a similar age, body condition, and diet that did not receive antibiotics (sample ID ASW29).

This type of individual variation was also evident when examining bacterial genera that were not as prevalent across the dataset but tended to have high relative abundances in some individuals. One individual, a domestic long-haired cat, had an anal gland where *Porphyromonas gingivalis* constituted 13% of the community (Fig. 1C, sample ID ASW68). In all other samples, this species had a relative abundance of < 0.5%. The anal gland of a male 6-year old cat with periodontal disease (sample ID ASW54) harbored the highest relative abundances of *Tesarococcus* sp. (23%), *Helcococcus kunzii* (8%), and *Lawsonella clevelandensis* (4%), whereas these taxa were < 2% abundant in the remaining cats (Fig. 1C). *Enterococcus* (mainly comprised by *E. avium*) constituted 4% of the anal gland microbiome in an 8-year old cat with lymphoma (sample ID ASW7), and this bacterial group was virtually absent from the anal glands of other cats (Fig. 1D). The anal glands of the smallest cat in the dataset (sample ID ASW20) which ate only canned food, housed the largest relative abundances of the butyric-acid producing bacteria *Butyricimonas virosa* (3%) and of *Prevotella copri* (4%) (Fig. 1D).

**Figure 2:**
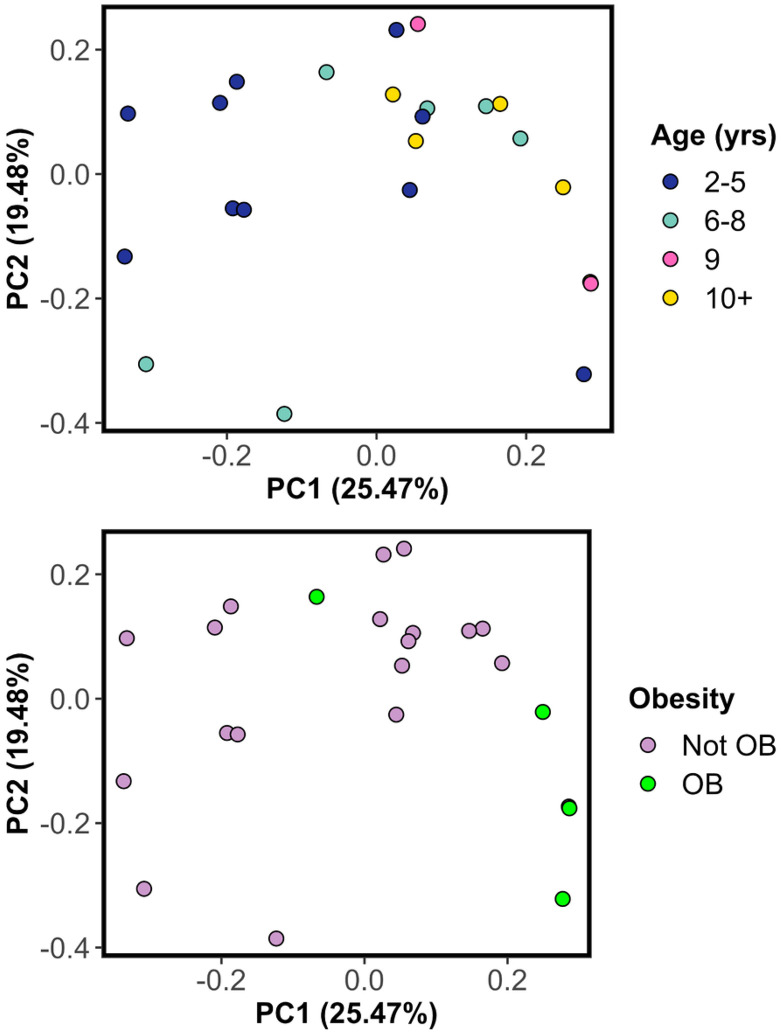
Microbiomes from the anal gland are significantly correlated with host age and obesity category. PCoA plots were constructed from Jaccard dissimilarity matrices based on Kraken2 genus-level relative abundances estimated from shotgun Illumina sequence data. Each point represents a sample and is color-coded by cat age in years (top) or obesity category (bottom). OB - obese. Closeness of points indicates high community similarity. The percentage of variance accounted for by each principal-coordinate axis is shown in the axes labels.

Next we asked whether any of the observed variation in bacterial community composition could be explained by host characteristics, including age (yrs), obesity (obese vs. not), living environment (indoor vs. indoor-outdoor), diet (dry kibble only vs. other), and a medical diagnosis of periodontitis (yes vs. no). Anal gland microbiome beta-diversity was significantly associated with host age and obesity (Fig. 2, Table 2). Specifically, the anal gland microbiome compositions of older cats were generally distinct from those of younger cats, both when taking into account the relative abundances of bacterial genera or simply their presence or absence (Table 2). The anal gland microbiome of obese cats was different compared to that of non-obese cats in terms of the bacteria genera present but the two groups were not different when taking into account the relative abundances of all bacterial genera (Fig. 2, Table 2). Host age and obesity accounted for 12% and 8% of the variance in microbiome profiles, respectively.

### Do metabolite profiles correlate with microbiome profiles?

A total of 428 metabolites were detected using solid phase microextraction (**Table S9**), of these 37 (8.6%) were putatively identified. Among the identified metabolites were fatty acids (nonanoic acid, hexadecanoic acid), esters (2-methylbutanoic acid, benzoic acid ethyl ester, pentanoic acid 4-methyl-), aldehydes (benzaldehyde, propanal), ketones (acetophenone, cyclohexanone), and hydrocarbons (ethylbenzene). The putatively identified compounds with the largest average relative abundances across samples after scaling were the aromatic hydrocarbons styrene and ethylbenzene and the aldehyde benzaldehyde.

For the liquid phase extractions, a total of 145 metabolites were detected, of which 51 (35.1%) were tentatively identified (**Table S10**). The derivatized samples contained cholesterol-related compounds, alcohols, and esters. The most abundant metabolites after scaling were the ester diethylene glycol dibenzoate, the benzoic-acid containing compound Oxybis(propane-1,2-diyl) dibenzoate, and the amine bis(tert-butyldimethylsilyl)amine. The low number of putatively identified volatile organic compounds (VOCs) reflects a limitation of the libraries used to identify compounds, in that they are not as exhaustive with regards to bacterial or fungal-associated VOCs.

**Figure 3:**
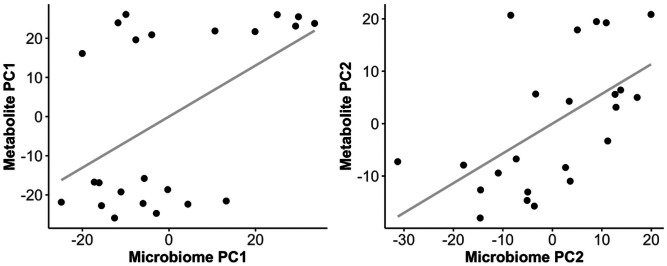
Microbiome profiles covary with metabolite profiles in the felid anal gland. Microbiome taxonomic profiles were estimated from shotgun Illumina sequence data with Kraken2/Braken and metabolite data were obtained using GC-MS with solid-phase microextraction. According to mantel tests, microbiome dissimilarity (Aitchison distance) was significantly correlated with metabolite dissimilarity (Euclidean distance). Principal coordinates were estimated for each data type and plotted. The gray line indicates the relationship between x and y as a linear model function.

Our next analysis determined whether microbiome profiles were significantly correlated with metabolite profiles. This would indicate that a relationship exists between the bacteria residing in the anal gland and the metabolites found in the anal gland. Microbiome and metabolome profiles detected during solid-phase microextraction were modestly positively correlated when using Aitchison distance for microbiome data (Mantel test r = 0.17, p = 0.01) but not when using Bray-Curtis distance (Mantel test r = 0.05, p = 0.23) or Jaccard distances (Mantel test r = 0.05, p = 0.26).This indicates that to an extent, samples with similar microbiome communities also tended to have similar VOC profiles (Fig. 3). Microbiome profiles were not significantly associated with metabolome profiles estimated after liquid derivatization (Mantel test Jaccard r = 0.09, p = 0.21; Bray-Curtis r= −0.04, p = 0.71; Aitchison r= −0.003, p = 0.51). Additionally, metabolome profiles did not vary with host characteristics for both the solid-phase and liquid-phase extractions (Table 2).

Next, we ascertained whether the relative abundances of specific bacterial species were significantly associated with the relative abundances of specific metabolites detected during solid-phase extractions since no relationship was found between microbiome profiles and metabolite profiles after liquid derivatization. For this, we regressed the relative abundances of 38 bacterial species which were present in 90% of samples at a mean relative abundance of at least 0.2% with the relative abundances of all 37 identified metabolites and 187 unidentified compounds (that all had mean relative abundances greater than zero after normalizing, log10-transforming, and scaling the raw values).

Two bacterial species had relative abundances that were correlated with the most metabolites, and many of those correlations were negative correlations with unidentified metabolites (**Table S15**). These bacteria were *Clostridioides perfringens* and *Streptococcus equi*. The relative abundances of five bacterial species were positively correlated with nonanoic acid – a fatty acid, and these taxa were two *Clostridium* species, *Fenollaria sporofastidiosus*, and two *Streptococcus* species. Eight bacterial species had relative abundances that were positively correlated with the relative abundances of a branched-chain fatty acid ester (2-methylbutanoic acid), including two *Clostridium* species, three *Streptococcus* species, *Ezakiella coagulans, Ezakiella massiliensis*, and *Fenollaria sporofastidiosus*. Sixteen percent of the bacterial species tested (6 out of 38 taxa) were positively correlated with two unidentified metabolites: NA_1277, NA_888.219, and negatively correlated with unidentified metabolite NA_2043 (**Table S15**). *Clostridium septicum*, and *Fenollaria sporofastidiosus* were negatively associated with the relative abundances of epicholestanol and cholesterol.

### Are microbiome putative functions related to VOC synthesis?

In addition to examining the composition of the microbiome and metabolome in the anal gland, we also inspected the predicted metabolic functional repertoire of these microbiomes and identified putative functions involved in the synthesis of VOCs. For this, we annotated genes predicted from metagenome contigs against the Cluster of Orthologous Genes (COG) and the Kyoto Encyclopedia of Genes and Genomes (KEGG) databases. For both datasets, we obtained annotations of specific genes (COG functions; KEGG orthologs) (**Table S12, Table S14**) and of predicted broader metabolic pathways (COG categories; KEGG modules) (**Table S11, Table S13**). Overall, 70.35% of host-filtered reads could be mapped to putative ORFs (range: 46–79%), and of these mapped reads, 66% (range: 50–71%) could be assigned COG annotations. This number was slightly lower for KEGG annotations (average 46%; range 31–58%) (**Table S2**).

Analysis of the most abundant COG functions or KEGG orthologs (**Table S12, Table S14**) across the dataset are not informative, as they mainly code for conserved metabolic functions related to bacterial growth and replication. A large percentage of predicted genes also code for putative transposases of undetermined function. To glean any functional insight, we instead mined the list of COG and KEGG annotations for any genes related to fatty-acid, aldehyde, ketone, or alcohol metabolism, regardless of their relative abundances in samples. It is important to note that alcohols can be *oxidized* into aldehydes or ketones, which can be further oxidized into carboxylic acids (fatty acids) and esters. Likewise, carboxylic acids can be *reduced* into aldehydes and ketones, and further reduced into alcohols. They can also be reacted with alcohols to make esters. This is just to say that the compounds are related to each other and share enzymatic machinery.

In COG profiles, we detected genes predicted to code for alcohol dehydrogenases, which oxidize alcohols into aldehydes and ketones, and aldehyde dehydrogenases, which oxidize aldehydes into carboxylic acids like acetic, propionic or valeric acid (**Table S10**). These same enzymes are also involved in the reverse reactions i.e. reducing fatty acids, aldehydes, or ketones into alcohols. Butanol dehydrogenase for example, catalyzes the conversion of butyraldehyde to butanol and was detected in the dataset. Putative genes also coded for alcohol-forming fatty acyl reductases which catalyze the reduction of thioesters to alcohols and are key enzymes for the microbial production of fatty alcohols. Microbes in the anal gland also contained genes predicted to encode acetyl CoA acetyltransferases, which are one of several proteins involved in the oxidation of fatty-acids into ketone bodies (like acetoacetate and beta-hydroxybutyrate). Acetoacetate decarboxylases which convert acetoacetate into acetone (a ketone) were also represented in the dataset (**Table S12**). Similarly, we detected KEGG orthologs predicted to code for dehydrogenases, decarboxylases, and reductases (**Table S14**).

**Figure 4:**
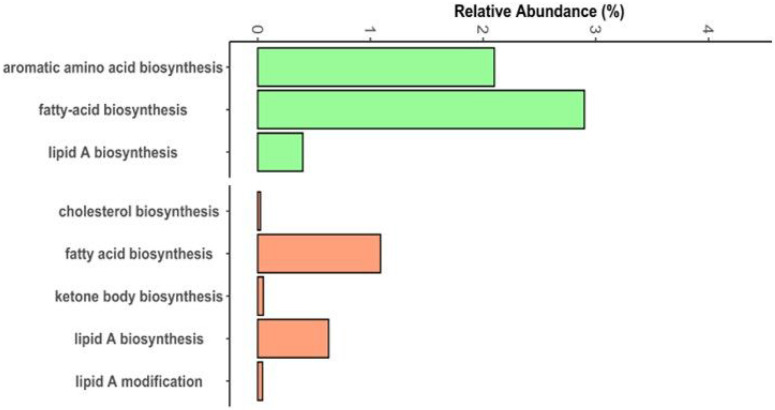
Relative abundance of microbial gene pathways potentially involved in VOC synthesis. Metagenome assemblies were functionally annotated with Anvi’o against the Cluster of Orthologous Genes (COG) or Kyoto Encyclopedia of Genes and Genomes (KEGG). The abundance of shotgun Illumina sequence data mapping to each pathway was calculated with Salmon in TPM and converted to relative abundances for analysis. Here we show the mean relative abundances of the three COG (green) and five KEGG (orange) pathways most relevant to VOC metabolism.

At a broad level, there were also bacterial pathways that encompassed functions relevant to the synthesis of volatile compounds. Out of 69 total COG pathways in the dataset, three were relevant: “fatty-acid biosynthesis”, “lipid A biosynthesis”, and “aromatic amino acid biosynthesis” (Fig. 4). Fatty acid biosynthesis was the fifth most abundant pathway and aromatic synthesis was the 13th most abundant (**Table S11**). The KEGG modules with most direct relevance to VOC metabolism were: ketone body biosynthesis, cholesterol biosynthesis, fatty acid biosynthesis, and lipid A biosynthesis and modification. The pathways were not super abundant in anal gland functional profiles (Fig. 4) (**Table S13**).

Next, we examined whether microbiome functional profiles were significantly associated with host factors or metabolite abundances. We found that anal gland microbiome functional repertoires were significantly correlated with host age and living environment. Host age accounted for 9–13% of the variance in functional microbiomes while host environment explained 11% of the variance (Table 2). Thus, slight differences in bacterial functions existed between the microbiomes of indoor cats and those of cats that lived indoors and outdoors. This distinction was not apparent when examining the abundances of broad functional pathways (**Table S16**). Furthermore, COG functional profiles (but not KEGG profiles) were moderately correlated (r = 0.11) with metabolite profiles acquired during solid-phase extraction (**Table S17**). That is, samples that were similar in their COG functional abundances tended to be somewhat similar in their metabolite abundances as well. This indicates that putative gene functions may be linked to putative metabolite abundances, and further work can pinpoint more direct links between specific microbial functions and metabolites. No significant or meaningful relationships were observed between the relative abundances of specific functional pathways and specific metabolites (data not shown).

### Reconstruction of metagenome-assembled genomes from potential bacteria of interest

Thus far, we provided a survey of the bacterial, functional, and metabolite composition of the anal gland microbiome in domestic cats. We tested whether variation was associated with host characteristics, or whether the relative abundances of specific bacterial genera covaried with metabolite relative abundances, in efforts to identify specific bacterial species or pathways that may be involved in microbial-mediated volatile synthesis. Analysis indicated that the most abundant genera in the anal gland were *Corynebacterium, Bacteroides, Proteus, Lactobacillus, Streptococcus*, and *Peptoniphilus*. The relative abundances of *Clostridium septicum, Fenollaria sporofastidiosus*, and *Streptococcus equi* were positively correlated with nonanoic acid (a fatty acid) while the relative abundances of *Ezakiella massiliensis*, and *Streptococcus anginosus*, among others, were positively correlated with butanoic acid 2-methyl (a branched-chain fatty acid ester). Next, we investigated whether genomes from any of the aforementioned bacterial taxa were reconstructed from shotgun sequencing data.

**Figure 5:**
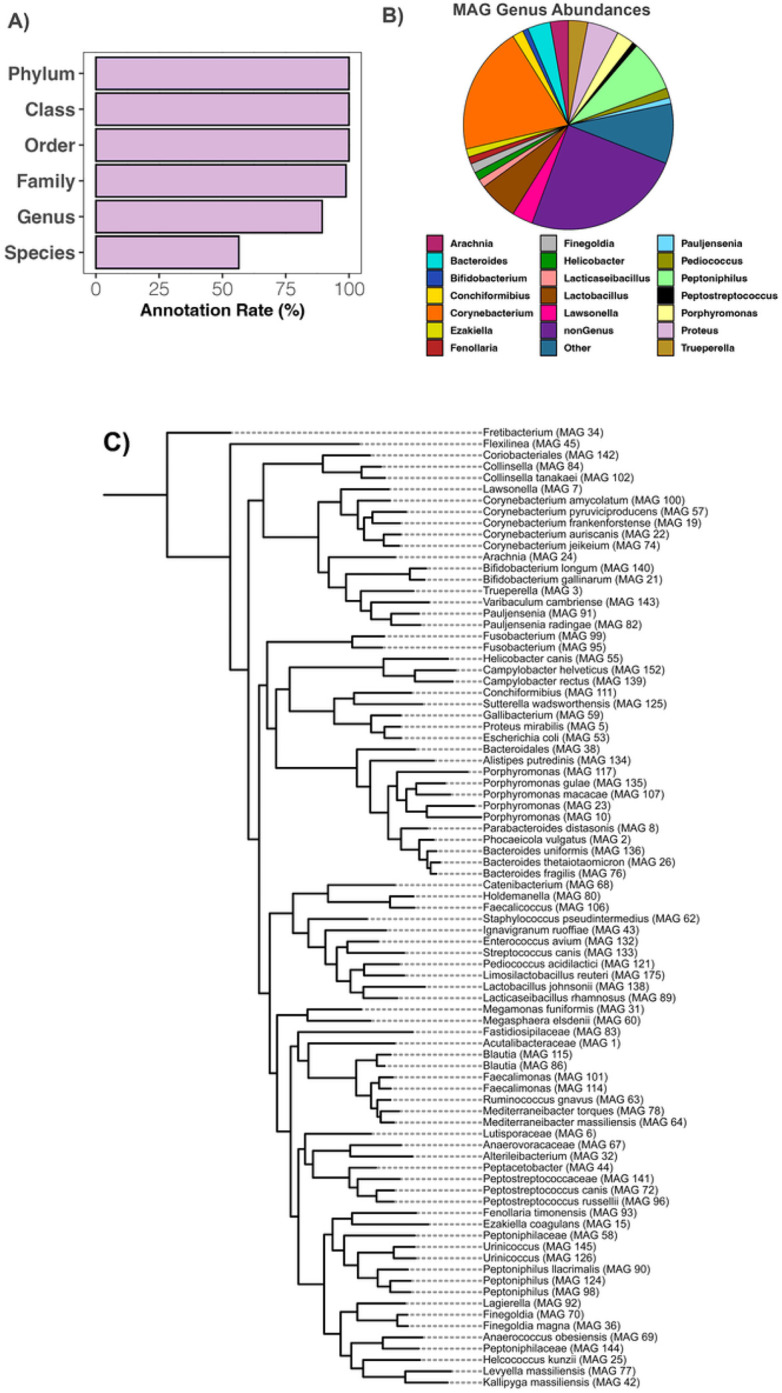
High-quality metagenome-assembled genomes (MAGs) reconstructed from the anal gland microbiome of domestic cats. Illumina shotgun sequence data were assembled into contigs with metaSPADES and binned into MAGs using MetaBat2. MAGs were assigned taxonomy with GTDB release 202 and MAGs with >80% completeness and <5% contamination were retained. **A)** Annotation rate for the 85-quality MAGs calculated by dividing the number of MAGs classified at that taxonomic level by the total number of MAGs. **B)** Average relative abundances of bacterial genera among MAGs, based on the number of Illumina shotgun sequencing reads that mapped to each MAG genus. The ‘nonGenus’ pie slice is the summed mean relative abundances of MAGs that did not receive a Genus classification. **C)** Phylogeny of MAGs constructed with alignments from GTDB. We had no outgroup for our phylogeny but rooted our tree to *Fretibacterium*, the only member of the phylum *Synergistota*.

A total of 85 high-quality metagenome-assembled genomes (MAGs) were recovered from the Illumina shotgun sequence data of the anal gland. These were on average 93.65% complete, and < 1% contaminated (Fig. 5C, **Table S4**). Thirty-five bacterial families, fifty-three genera, and forty-seven species were represented by one or more MAGs. Close to 90% of MAGs were classified to genus level and over half (54%) were classified to species level (Fig. 5A). On average, 60.37% of Illumina shotgun sequences in each sample were aligned to the MAGs (range: 32–88%).

Of the portion of Illumina shotgun metagenomic reads that were aligned to MAGs, 19.8% of those reads were from MAGs classified to the genus *Corynebacterium* (Fig. 5B, **Table S5**). This number is highly comparable to that obtained from analyzing the taxonomic composition of Illumina shotgun reads (e.g. Kraken genus-level abundance data), where the abundance of *Corynebacterium* was 18%. Other bacterial taxa with high relative abundances in both MAGs and metagenomes included *Peptoniphilus* (7.9% MAG mean relative abundance vs. 4% mean relative abundance in the Kraken metagenome dataset), *Lactobacillus* (6.08% vs. 7% in metagenome dataset), and *Proteus* (4.83% vs. 7% in metagenome dataset). *Bacteroides* was much more represented in the metagenome dataset (11% mean relative abundance) than as MAGs (3.5% mean relative abundance) (Fig. 5B). The genus *Lawsonella* (3.22% mean relative abundance) was similarly represented in MAGs compared to the metagenome dataset (2.66%).

The six MAGs with the highest mean relative abundances across anal gland metagenomes were: MAG #144 *Peptoniphilaceae* (22.5%), MAG #19 *Corynebacterium frankenforstense* (8.9%), MAG #124 *Peptoniphilus* (6.5%), MAG #138 *Lactobacillus johnsonii* (6.08%), MAG #57 *Corynebacterium pyruviciproducens* (5.39%), and MAG #5 *Proteus mirabilis* (4.8%) (**Table S5**). All other MAGs were found at mean relative abundances of < 4%. The *Peptoniphilaceae* MAG was most closely related to genomes belonging to several *Anaerococcus* species in the Genome Taxonomy Database release 202 (**Fig S2**). The closest relative to MAG #19 was a *C. frankenforstense* isolated from raw cow’s milk. The *Peptoniphilus* MAG #124 is evolutionarily related to a *P. lacydonensis* isolated from the human sinus (**Fig S2**). The closest relatives to the remaining MAGs were microbes from the same Genera isolated from the human body as part of the Human Microbiome Project.

**Figure 6:**
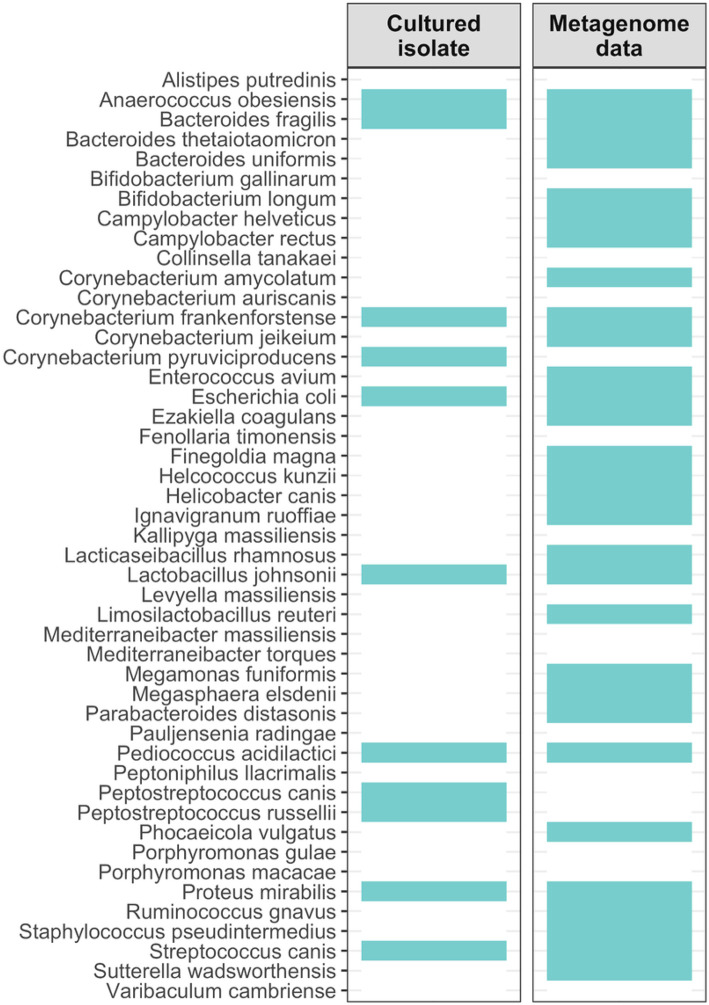
Comparing MAGs, cultured isolates, and metagenome profiles. We investigated whether bacterial species that were represented by one or more MAGs (a total of 47 taxa) were also recovered in the lab as cultured isolates and were present in Kraken2 metagenome profiles that were based on Illumina shotgun reads. Turquoise cells indicate that the bacterial taxon was represented by a bacterial isolate and was present in the metagenome dataset, while a grey cell indicates the opposite. It is important to note that MAGs, cultured isolates, and metagenomic reads were assigned taxonomic labels using different databases.

A total of 11 bacterial species for which we had quality-MAGs also had cultured isolates from this study and were present in the larger metagenome dataset (Fig. 6, **Table S18**). These bacterial species were: *Streptococcus canis, Proteus mirabilis, Pediococcus acidilactici, Lactobacillus johnsonii, Escherichia coli, Corynebacterium frankenforstense, Bacteroides fragilis*, and *Anaerococcus obesiensis* (Fig. 6). Of these species, the 4 with the highest relative abundances in the microbiome dataset were *C. frankenfortstense* (9.9% mean relative abundance), *P. mirabilis*) (7.1%), *L. johnsonii* (6.4%), and *B. fragilis* (5.5%) (**Table S8**). These four bacterial species make good candidates for further investigation into their potential contributions to fatty acid and volatile compound production.

Analyses showed that MAG relative abundances in the anal gland varied with host age, which accounted for 8–10% of the variance (Table 2). Furthermore, the anal glands of indoor house cats did not contain all of the same MAGs as the anal glands of cats that had access to both the indoors and outdoors (Table 2). The remaining predictors, including obesity category, diet, and a medical diagnosis of periodontitis did not significantly predict MAG abundances. MAG abundance profiles were not correlated with metabolite profiles acquired during solid-phase extraction (Mantel Jaccard r = 0.03, p = 0.34; Bray-Curtis r = 0.06, p = 0.17; Aitchison r = 0.05, p = 0.2) or after liquid derivatization (Mantel Jaccard r = 0.05, p = 0.25; Bray-Curtis r=−0.06, p = 0.8; Aitchison r = 0.004, p = 0.44).

Are anal gland microbiomes distinct from perianal microbiomes? Because microbiome data from both the perianal region and anal gland were available for six cats, we tested whether the two body-sites harbored different microbiomes. According to PERMANOVA tests, microbiomes from the anal gland did not necessarily contain all of the same microbes that were found in the perianal region (Jaccard index R^2^ = 0.14, p = 0.03), although the two body sites were not distinct when taking into account the relative abundances of all bacterial taxa (Bray-Curtis R^2^ = 0.10, p = 0.14; Aitchison R^2^ = 0.09, p = 0.14). There was also evidence of host-specificity in the microbiome, as host identity accounted for 55–60% of the variation (Jaccard R^2^ = 0.55, p = 0.036; Bray-Curtis R^2^ = 0.49, p = 0.13; Aitchison R^2^ = 0.60, p = 0.01). This also shows that there is consistency between the composition of the microbiome in the anal gland and the microbiome in the perianal region within individuals.

Plots of microbiome composition indicate that abundances of dominant bacterial genera are distinct in the anal gland compared to the perianal region (**Fig S1**). Microbiomes of the anal gland appeared to be enriched in *Corynebacterium* (mean relative abundance of 16.98% in the anal gland vs. 2.70% in perianal microbiomes) and *Lactobacillus* (mean relative abundance of 15.8% in anal gland vs. 0.87% in the perianal region). Perianal microbiomes instead contained greater abundances of *Collinsella* (mean relative abundance 4.45% in perianal microbiomes vs. 0.25% in anal gland microbiomes), *Escherichia* (mean relative abundance 10.8% vs. 0.75%), and *Helicobacter* (mean relative abundance 8.31% vs. 1.91%) (**Fig S1, Table S8**).

The two body-sites did not differ in the abundances of their metagenome-assembled genomes (PERMANOVA Bray-Curtis R^2^ = 0.07, p = 0.35; Aitchison R^2^ = 0.07, p = 0.89). But did vary in the MAGs they contained (PERMANOVA Jaccard R^2^ = 0.05, p = 0.006). Lastly, the functions encoded by the perianal and anal gland microbiomes were not fundamentally different at broad levels (**Table S19**), but were different in terms of the presence/absence of specific COG functions and KEGG orthologs (**Table S19**). As echoed earlier, host identity also explained a significant amount of functional variation (51–60%) in anal gland and perianal microbiomes (**Table S19**).

## Discussion

The main purpose of this study was to survey the bacterial, functional, and metabolite composition of the anal gland microbiome in 23 companion cats in efforts to identify bacterial taxa or gene pathways potentially involved in the synthesis of volatile organic compounds (VOCs) being used by the host during chemical signaling. We also add to the limited literature on the anal gland microbiome and metabolome of cats.

### Microbiome composition of the anal gland in felines compared to other scent-producing mammals

Our work demonstrated that the bacterial genera with the highest relative abundances in the anal gland of domestic cats were *Corynebacterium, Bacteroides, Proteus, Lactobacillus, Streptococcus*, and *Peptoniphilus*. This is distinct from what has been reported for the anal glands of a Bengal cat (*Felis catus × Prionailurus bengalensis*), where 95% of the microbiome community was comprised by *Tessaracoccus, Anaerococcus*, and *Finegoldia* [37]. These differences might be attributed to host species differences (domestic cat vs. Bengal cat) or study methodologies (e.g. amplicon sequencing in the Bengal cat study vs shotgun sequencing here). The anal gland microbiomes of domestic dogs, however, harbored relative abundances of *Bacteroides* and *Proteus* similar to what was found in the surveyed cats [44]. In red foxes, *Proteus mirabilis* is consistently isolated from anal gland secretions, but not always detected in fecal samples [79]. Similar to cats, the anogenital gland secretions of giant pandas are dominated by *Corynebacterium* (and *Pseudomonas*, and *Porphyromonas*) [38]. In the musk secretions of Chinese forest deer, *Corynebacterium* was the most abundant bacterial genus [80]. The anal gland microbiome of the surveyed cats however did not mirror that found in wild spotted hyenas (*Crocuta crocuta*), where 95% of sequences were classified as *Clostridiales (Anaerococcus, Clostridium, Fastidiosipila, Finegoldia, Peptoniphilus*, and *Tissierella*) [21]. This could be due to species differences in diet, habitat, physiology, social behavior, and social structure.

The anal gland microbiomes of the domestic cats were also differentiated by host age and obesity category (obese vs. not obese). The cats in our study were all adults and varied in age from 2–14 years old. Age-specific differences in the microbiome could be due to physiological, immunological and behavioral changes experienced by cats as they age. Compared to younger cats, older cats – particularly those 10 years of age and older – may experience reduction in their sense of smell, hearing and vision [81]. Aging cats may also experience thinning of the hair coat, deterioration of their joint components, and reduced nutrient digestibility and absorption [81]. They may have suppressed immunity. Thus, it is not surprising that such changes are associated with different microbiome compositions in the anal gland. Regarding obesity, no prior studies conducted in other scent-producing mammals have examined differences in the scent gland microbiome of obese and non-obese individuals. The fecal microbiomes of domestic cats however, do vary between obese and non-obese individuals [82, 83]. Systemic health conditions associated with feline obesity, including insulin resistance, urinary disease, and cardiovascular disease [84] – may underline microbiome changes. Lastly, the anal gland microbiomes of the surveyed felines showcased tremendous amounts of individual variability, echoing what has been found for domestic dogs [44].

Felid anal gland microbiomes did not vary between individuals diagnosed with periodontitis and those not diagnosed with the disease. Periodontal disease which involves inflammation and recession of the gums [85] can cause oral tissue loss, oral discomfort and pain, and may even impact the animal’s longevity and quality of life [86]. Although severe, these changes may not impact the feline body in a way that would cascade to changes in the anal gland, a very distal part of the body. The anal gland mainly interacts with apocrine and sebaceous glands, neighboring rectal sphincter muscles, and subcutaneous tissue and skin [87]. If those areas are affected such as when cats experience anal gland abscesses or inflammation [88], it may be more likely to observe changes in the anal gland microbiome composition. Interestingly, we did find evidence that the microbiome functional repertoires in the anal gland were associated with host living environment; cats that lived indoors had microbiome functions that were not identical to cats that had outdoor access. It is thought that cats with outdoor access are more likely to be infected with parasites than indoor-only cats [89] but may have increased exposure to natural enrichment and mental stimulation. Conversely, cats that have an indoor-only lifestyle may experience reduced physical activity, greater food consumption, and less natural enrichment [90]. Nonetheless, it is not clear how these lifestyle differences and contact with the outdoors may cause or be a result of functional differences in the anal gland microbiome.

### Metabolite composition of the anal gland in felines compared to other scent-producing mammals

The classes of metabolites detected in the anal gland of companion cats were consistent with those previously found in the glandular secretions of European badgers [33], red foxes [91], meerkats [39], domestic dogs [92], coyotes [93], giant pandas [38], forest musk deer [80], bearded emperor tamarins [94], and owl monkeys [43]. Similar to cats, the glandular microbiomes of these mammalian species also contained volatile compounds such as aldehydes, hydrocarbons, fatty acids, ketones, esters, alcohols, or aromatic compounds.

Although the chemical composition of scent gland secretions were not identical between the surveyed cats and Bengal cats [37], other domestic cats [53], or domestic dogs [92], they did overlap in regards to certain compounds. The anal glands of other domestic cats and the cats in this study both contained butanoic acid, methylbutanoic acid, and pentanoic acid [53]. Compared to dogs, the anal glands of cats also contained benzene compounds, phenols, and several fatty acids (pentanoic, butanoic, and pentadecenoic acids) [92]. Butanoic acid like pentanoic acid is a short chain fatty acid and a major intermediate in the anaerobic degradation of organic compounds. It has a strong sweet rancid odor and is also a constituent of fox [95], pig [96], and human body odors [97].

Compounds such as indole, xylene, hexadecanoic acid, and nonanoic acid were detected in the anal glands of both Bengal cats [37] and the cats in our study. Xylene is a cyclic hydrocarbon that is ubiquitous in decomposed animal tissue [98], and has been found in the soil and air of animal farms [99]. With the exception of surveys of the anal gland in Bengal cats [37] and giant pandas [38], xylene has not been widely reported in the glands of other canids, mustelids, or primates. Furthermore, because xylene is also an environmental pollutant and is used in dyes, paints, and industry solvents [100], its direct relevance to chemical communication in domestic cats is unknown. Indole which is an aromatic compound produced by the bacterial deamination of the amino acid tryptophan appears to be enriched in the feces of sick hedgehogs which attract ticks more than the feces of healthy hedgehogs [101]. Indole has also been recovered from the skin and feces of reticulated giraffes [102], volatile headspace of human sweat [103], and sewage or animal waste [104]. This compound is an essential metabolite involved in plant-insect interactions and is emitted as a scent by plants to attract pollinators [105]. These lines of evidence suggest that indole may have some significance to odor composition and chemical signaling in cats and other mammals.

### Relationships between microbes and metabolites in the anal gland

Although every single compound found in the glandular secretions of cats cannot be explicitly linked to host odor and chemical communication, our study did find that the metabolome profiles were overall correlated with microbiome profiles. This finding suggests that a link exists between the metabolites in the anal gland and the microbes in the anal gland. This finding supports prior work conducted in meerkats [39], giant pandas [38], striped and spotted hyenas [27], and European badgers [33]. In Bengal cats an even more direct link exists between the anal gland microbiome and its metabolites given that bacteria cultured from the anal gland produced many of the same volatiles detected in anal gland secretions [37]. Furthermore, in song sparrows, preen gland microbiota does not correlate with its chemistry but does covary with host MHC (major histocompatibility complex) genotype [106], which is implicated in mate choice and may influence odor directly or indirectly. Experimental evidence that microbes directly contribute to chemical signaling is also documented in humans, where microbes isolated from the skin can produce volatile compounds that attract African malaria mosquitos [107]. These inter-domain interactions reveal that microbes are producing volatile compounds that can be recognized by their vertebrate hosts.

In our study, the relative abundances of several anal gland bacteria were positively correlated with the relative abundances of two putatively identified metabolites (and several unidentified metabolites). Bacteria from the genus *Clostridium, Streptococcus, Fenollaria*, and *Ezakiella* were positively correlated with the relative abundances of 3-methylbutyric acid, a fatty acid ester also known as isovaleric acid. Isovaleric acid has been recovered from the anal glands of dogs [92], coyotes [93], and red foxes [91]. Two enzymes – a transaminase and a decarboxylase – are involved in its synthesis and have been purified from *Clostridium bifermentans* [108]. Isovaleric acids have also been recovered from cultures of the following *Clostridium* species: *C. difficile, C. bifermentans, C. sporogenes, C. subterminalis*, and *C. putrefasciens* [109]. Thus, the genetic machinery for isovaleric acid production may exist in one or more *Clostridium* species.

The second positive association was between the relative abundances of nonanoic acid and the relative abundances of six bacterial species from the genera *Clostridium, Fenollaria*, and *Streptococcus*. Again this is significant because correlations with the remaining 35 identified metabolites were not statistically robust. Nonanoic acids were one of six carboxylic acids that impacted the aroma of yogurt fermented by *Streptococcus thermophilus* [110]. These fatty-acids can be produced from the oxidation of nonanal – an aldehyde– via the activity of aldehyde dehydrogenases (ALDH). NADP-dependent ALDH have been characterized in *Streptococcus mutans* [111], indicating that *S. mutans* and relatives have the functional capacity to produce nonanoic acid and other carboxylic acids. It is important to note that we identified several putative genes in the larger microbiome dataset that were predicted to code for aldehyde or alcohol dehydrogenases, fatty acyl reductases, and decarboxylases which can collectively oxidize or reduce compounds that are constituents of mammalian scent.

The relative abundances of two bacterial species – *Clostridium septicum*, and *Fenollaria sporofastidious* – were negatively correlated with the relative abundances of epicholestanol and cholesterol, indicating they consume cholesterol compounds or interact antagonistically with bacteria that synthesize those compounds. Cholesterol is found in animal cell membranes and is required for steroid hormone, vitamin D, and bile acid synthesis [112]. It can be oxidized into cholesterol aldehydes and has been found in high relative abundances in the anal glands of giant pandas [38] and alpine marmots [30]. Nonetheless, its relevance to host odor and chemical communication in cats is not clear.

### Four potentially important bacterial species of the felid anal gland

To our knowledge, we are the first to present high-quality metagenome assembled genomes (MAGs) from the anal gland of cats and companion animals in general. We call attention to four MAGs in particular that were abundant in the larger microbiome dataset and were recovered as cultured isolates: *Corynebacterium frankenforstense, Proteus mirabilis, Lactobacillus johnsonii*, and *Bacteroides fragilis*. These four bacterial taxa represent candidates for further study into their contributions to mammalian scent and chemical communication.

Prior studies show that *Corynebacteria* are common inhabitants of the anal glands [113], perineal glands [114], musk glands [80], and axillae [115] of mammals. Researchers performing both correlative and experimental studies report that *Corynebacteria* can cleave odorant precursors present in the human armpit which leads to the release of short-branched fatty acids that are key components of axillary odor [116].

*P. mirabilis* isolated from the salivary gland of blow fly maggots secrete indoles, carboxylic acids, phenols, and putrescine compounds that attract blowflies to animal carcasses [117]. *P. vulgaris*, a close relative of *P. mirabilis*, produces the largest number of aromatic compounds (e.g. esters, ketones, aldehydes, alcohols, and sulfides) during cheese ripening out of any other bacteria present in French cheese rinds [118]. Via enzyme activity assays, *P. mirabilis* have also been shown to possess fatty acid decarboxylases and alcohol dehydrogenases, which are required for VOC synthesis [119, 120].

*Bacteroides fragilis* isolated from the anal gland of a Bengal cat produced the same volatile compounds present in anal gland secretions [37]. *Bacteroides* spp., which are one of the most abundant gram-negative bacteria in the human gut, are well-known producers of short-chain fatty acids including acetic, isobutyric, propionic, isovaleric, and succinic acids [121].

Although *Lactobacillus* spp. are not typically found in anal gland secretions (with the exception of European badgers [33]), they can ferment sugars to produce lactate, acetate, or ethanol; the latter two which are anal gland volatile compounds [122]. Lactic acid (the nonaqueous form of lactate) is detected in the perineal glands of North American porcupines [114] and tarsal glands of white-tailed deer [123]. Furthermore, in the harlequin ladybird beetle, *Lactobacillus* spp. produce volatiles that function as important semiochemicals during host antipredatory defense behavior [124]. Lastly, in Wistar rats, tetradecanoic acid – a saturated fatty acid– was found to be lower in concentration in socially stressed rats, but administration of *Lactobacillus paracase*i via oral gavage prevented its depletion and improved anxiety-like behavior [125]. These lines of evidence suggest that *Lactobacillus* spp. are able to synthesize volatile organic compounds that function as cues and affect mammalian behavior.

### Establishing ranges of healthy anal gland microbiomes

The cats that participated in our study had underlying dental and intestinal disorders, but their anal glands were healthy (no abscesses, tumors, or infections) and thus, our study showcases variation present in the microbiome of healthy anal glands. We show that there is tremendous individual variation in the anal gland microbiomes of domestic cats and the microbiome does not have a particular composition, which will be important to consider when making comparisons to infected anal glands. Nonetheless, several species of bacteria were consistently present and abundant in the anal glands of our cohort of cats, among them *Proteus spp.*, and *Corynebacterium spp*. Our work can contribute to the larger body of work aiming to detect deviations and abnormalities in anal gland microbiomes to devise effective therapeutic treatments that restore the microbiome balance. We share high-quality MAGs from the felid anal gland which can be further characterized and annotated to determine whether they have gene pathways or metabolisms of relevance to anal gland health or treatment.

### Limitations

This was primarily a descriptive study that reported on the variation present in the microbiome and metabolome of the anal gland in cats, in an effort to highlight bacterial species or functions potentially involved in VOC synthesis and host odor production. No direct experimental evidence is provided and future studies will be needed to test the associations and patterns observed in a controlled experiment. Secondly, our sample size of twenty-three cats is small and thus findings only apply to a small group of animals. We encourage future studies to conduct a larger-scale analysis of the anal gland microbiome and metabolome in cats to gain additional biological insights on the anal gland microbiome of felines. As mentioned above, the cats in our study were clinically healthy and had healthy anal glands, but had been previously diagnosed with diseases that affected other areas of the body. This is something to keep in mind for future studies that want to compare their findings to ours. Despite these limitations, our study fills in a large gap in the literature as only few studies have examined the microbes or metabolites in the anal gland in cats. We provide a combined analysis of the felid microbiome and metabolome in the anal gland and share metagenome-assembled genomes (MAGs) for this body site for this species which future work can build upon.

## Figures and Tables

**Table 1:** Characteristics of the twenty-three domestic cats (N = 23) that participated in this study.

Characteristic	Subcategory	Number of Cats (N = 23)
**Age, in years**		Range: 2–14 Median: 6.9
**Body condition (1–10)**	3–4	4 (17%)
	5–6	10 (43%)
	7	5 (22%)
	8–9	4 (18%)
**Sex**	Female	12 (52%)
	Male	11 (48%)
**Breed**	Domestic Shorthair	19 (83%)
	Other	4 (17%)
**Diet**	Dry Kibble only	13 (57%)
	Dry Kibble and Canned food	6 (26%)
	Other	4 (17%)
**Environment**	Indoor	16 (70%)
	Indoor & Outdoor	7 (30%)
**Antibiotics** (within prior 6 months)	Yes	1 (4%)
	No	22 (96%)
**Periodontal disease diagnosis** (moderate to severe)	Yes	9 (39%)
	No	14 (61%)
**IBD diagnosis**	Yes	3 (13%)
	No	20 (87%)

Samples were collected from the anal glands of twenty-three cats that were presented to the Veterinary Teaching Hospital at our institution for elective procedures that required sedation or general anesthesia. Health and lifestyle data on each individual cat was recorded and summarized above. The full list of samples and their metadata are provided in Table S1.

**Table 2:** Do host characteristics predict bacterial, metabolite, and functional pathway abundances in the anal gland?

Data type	Distance type	Age (yrs)	Obesity (obese vs. not)	Living environment (indoor vs. indoor outdoor)	Diet (Dry food only vs. Other)	Medical diagnosis (periodontitis)
Microbiome	Jaccard	**0.092 (p = 0.013)**	**0.087 (p = 0.034)**	0.054 (p = 0.18)	0.029 (p = 0.77)	0.024 (p = 0.9)
	Bray-Curtis	**0.12 (p = 0.003)**	0.029 (p = 0.74)	0.043 (p = 0.37)	0.042 (p = 0.43)	0.034 (p = 0.57)
	Aitchison	0.068 (p = 0.09)	0.041 (p = 0.47)	0.055 (p = 0.20)	0.054 (p = 0.22)	0.046 (p = 0.35)
Metabolome (solid-phase)	Jaccard	0.016 (p = 0.95)	0.017 (p = 0.91)	0.029 (p = 0.67)	0.057 (p = 0.27)	0.033 (p = 0.61)
	Euclidean	0.025 (p = 0.91)	0.028 (p = 0.86)	0.035 (p = 0.61)	0.06 (p = 0.2)	0.039 (p = 0.5)
Metabolome (liguid-derivatization)	Jaccard	0.051 (p = 0.33)	0.045 (p = 0.39)	0.057 (p = 0.26)	0.019 (p = 0.88)	0.035 (p = 0.57)
	Euclidean	0.044 (p = 0.41)	0.039 (p = 0.50)	0.061 (p = 0.17)	0.024 (p = 0.86)	0.037 (p = 0.54)
COG functions	Jaccard	0.049 (p = 0.24)	0.049 (p = 0.29)	**0.12 (p = 0.025)**	0.026 (p = 0.7)	0.021 (p = 0.79)
	Bray-Curtis	**0.13 (p = 0.005)**	0.045 (p = 0.32)	0.04 (p = 0.4)	0.038 (p = 0.44)	0.029 (p = 0.63)
	Aitchison	**0.085 (p = 0.01)**	0.045 (p = 0.32)	0.05 (p = 0.23)	0.041 (p = 0.43)	0.037 (p = 0.56)
KEGG functions	Jaccard	0.045 (p = 0.31)	0.045 (p = 0.33)	**0.11 (p = 0.039)**	0.022 (p = 0.8)	0.019 (p = 0.86)
	Bray-Curtis	**0.13 (p = 0.008)**	0.037 (p = 0.43)	0.045 (p = 0.29)	0.036 (p = 0.44)	0.028 (p = 0.64)
	Aitchison	**0.092 (p = 0.01)**	0.041 (p = 0.46)	0.05 (p = 0.21)	0.042 (p = 0.41)	0.035 (p = 0.61)
Metagenome-assembled genomes (MAGs)	Jaccard	0.051 (p = 0.23)	0.040 (p = 0.58)	**0.068 (p = 0.04)**	0.045 (p = 0.41)	0.045 (p = 0.4)
	Bray-Curtis	**0.10 (p = 0.004)**	0.038 (p = 0.49)	0.058 (p = 0.11)	0.041 (p = 0.47)	0.36 (p = 0.61)
	Aitchison	**0.085 (p = 0.014)**	0.027 (p = 0.91)	0.045 (p = 0.43)	0.036 (p = 0.7)	0.034 (p = 0.75)

Kraken2 Genus-level bacterial relative abundances were used to estimate microbiome Jaccard, Bray-Curtis or Aitchison dissimilarity distances. Metabolite GC-MS intensity values were normalized, log_10_-transformed and scaled to estimate Jaccard and Euclidean distances. Metagenome sequences mapping to putative gene or gene pathways were converted from Transcripts per Million to relative abundances to calculate Jaccard, Bray-Curtis or Aitchison matrices. The number of shotgun sequences mapping to MAGs was used to calculate relative abundances to construct the same three dissimilarity matrices. PERMANOVA models using 999 permutations correlated five host predictors with bacterial, metabolite, or functional beta-diversity. All predictors were evaluated simultaneously – in a way where the order of terms did not influence the statistical outcome. Significant p-values (a = 0.05) are bolded.

## Data Availability

Raw shotgun sequences have been uploaded to the NCBI Sequence Read Archive (SRA) (accession numbers SRR24332691-SRR24332721), under the BioProject PRJNA961122. The ASV relative abundance table, ASV taxonomic classifications, and corresponding sample metadata are available as supplementary materials. The R code for conducting all statistical analyses and generating all figures presented in this article is stored in a public GitHub repository (https://github.com/rojascon/Cat_AnalGland_Microbiome_Metabolome).
